# Carbapenem Resistance in Gram-Negative Bacteria: A Hospital-Based Study in Egypt

**DOI:** 10.3390/medicina59020285

**Published:** 2023-02-01

**Authors:** Amira Abd Elrahem, Noha El-Mashad, Mohammed Elshaer, Hazem Ramadan, Giovanni Damiani, Monir Bahgat, Santo Raffaele Mercuri, Wafaa Elemshaty

**Affiliations:** 1 Faculty of Medicine, Mansoura University, Mansoura City 35516, Egypt; 2 Clinical Pathology Department, Faculty of Medicine, Mansoura University, Mansoura City 35516, Egypt; 3 Hygiene and Zoonoses Department, Faculty of Veterinary Medicine, Mansoura University, Mansoura City 35516, Egypt; 4 Young Dermatologists Italian Network (YDIN), 20122 Milan, Italy; 5 Department of Biomedical, Surgical and Dental Sciences, University of Milan, 20122 Milan, Italy; 6 Ph.D. Degree Program in Pharmacological Sciences, Department of Pharmaceutical and Pharmacological Sciences, University of Padua, 35131 Padua, Italy; 7 Department of Dermatology, Case Western Reserve University, Cleveland, OH 2109, USA; 8 Internal Medicine Department, Faculty of Medicine, Mansoura University, Mansoura City 35516, Egypt; 9 Unit of Dermatology, IRCCS San Raffaele Hospital, 20132 Milan, Italy

**Keywords:** carbapenemase, plasmids, *Klebsiella*, *Escherichia coli*, Egypt, replicon

## Abstract

*Background and Objectives:* The global spread of carbapenem resistance and the resulting increase in mortality forced the World Health Organization (WHO) to claim carbapenem-resistant *enterobacteriaceae* (CRE) as global priority pathogens. Our study aimed to determine the prevalence of carbapenemase-encoding genes and major plasmid incompatibility groups among Gram-negative hospital-based isolates in Egypt. *Material and Methods*: This cross-sectional study was carried out at Mansoura University Hospitals over 12 months, from January to December 2019. All the isolates were tested for carbapenem resistance. The selected isolates were screened by conventional polymerase chain reaction (PCR) for the presence of carbapenemase genes, namely *bla_KPC_*, *bla_IMP_*, *bla_VIM_*, and *bla_NDM-1_*. PCR-based plasmid replicon typing was performed using the commercial PBRT kit. *Results*: Out of 150 isolates, only 30 (20.0%) demonstrated carbapenem resistance. *Klebsiella pneumoniae* was the most resistant of all isolated bacteria, and *bla_NDM_* was the predominant carbapenemases gene, while the most prevalent plasmid replicons were the F replicon combination (FIA, FIB, and FII) and A/C. Plasmids were detected only in *Klebsiella pneumoniae*, *Escherichia coli*, *Enterobacter cloacae,* and Pseudomonas *aeruginosa*. Remarkably, we found a statistically significant association between carbapenemase genes and plasmid replicons, including *bla_NDM_*, IncA/C, and IncX. *Conclusions*: Our study demonstrated an alarming rise of plasmid-mediated carbapenem-resistant bacteria in our locality. The coexistence of resistance genes and plasmids highlights the importance of a targeted antibiotic surveillance program and the development of alternative therapeutic options at the local and international levels. Based on our results, we suggest a large-scale study with more *Enterobacteriaceae* isolates, testing other carbapenemase-encoding genes, and comparing the replicon typing method with other plasmid detection methods. We also recommend a national action plan to control the irrational use of antibiotics in Egypt.

## 1. Introduction

The unrestricted access to antibiotics and empirical and preventive overuse, together with the antibiotic addition to animal food, may be responsible for the dramatic increase in drug-resistant bacteria, especially in hospitals [1].

Although all bacteria have the potential to develop antibiotic resistance, Gram-negative bacteria, particularly *Enterobacteriaceae* and *Pseudomonas,* are frequently implicated in hospital infections, representing a significant clinical and socioeconomic burden [2].

Since extended-spectrum β-lactamase (ESBL)-producing bacteria became a dramatic reality, this imposed a change in the first-line empirical treatment from penicillins to carbapenems [3,4].

The global spread of carbapenem resistance and the resulting increase in mortality forced the World Health Organization (WHO) to claim carbapenem-resistant *enterobacteriaceae* (CRE) as global priority pathogens [4,5,6,7].

Enzyme-mediated resistance to carbapenems is mediated by beta-lactamases that are capable of inactivating carbapenems and other beta-lactam antibiotics and are hence referred to as carbapenemases [5]. This type of resistance is the most relevant clinically because these enzymes are encoded by genes that are horizontally transferable by plasmids or transposons and are often associated with genes encoding other resistance determinants [6,7].

*Enterobacteriaceae* contain a wide range of carbapenemases from the three Ambler classes of β-lactamases: A, B, and D [8]. The *Klebsiella pneumoniae* carbapenemase gene (KPC), Verona integron-encoded metallo-β -lactamase gene (VIM), IMP-type metallo-β-lactamase gene (IMP), New Delhi metallo-β-lactamase gene (NDM), and oxacillinase group of β-lactamases gene (OXA-48) are the most potent carbapenemase-producing genes in terms of carbapenem hydrolysis and geographic spread [9].

Although several studies have been carried out on the detection of plasmid-mediated clinical isolates of *Enterobacteriaceae* strains worldwide [10,11,12,13], only a few reports have been published in Egypt [14,15].

Hence, we performed this study to determine the prevalence of carbapenemase-encoding genes and major plasmid incompatibility groups among Gram-negative hospital-based isolates in Egypt.

## 2. Material and Methods

### 2.1. Study Design

A cross-sectional observational study was conducted in the period between January 2019 and December 2019 at Mansoura University Hospitals. The study was compliant with the Helsinki Declaration, and all included patients signed informed consent forms. In this specific form, it was explicitly written that data derived from routine exams performed in the Mansoura University Hospitals could be used for research if anonymized.

### 2.2. Inclusion and Exclusion Criteria

In the present study we enrolled (a) adult patients (>18 year) who were (b) immunocompetent and (c) who signed the informed consent form. Likewise, we excluded (a) pediatric patients, (b) transplanted patients, (c) HIV-positive patients, and (d) patients undergoing chemotherapy, immunotherapy, or radiotherapy.

### 2.3. Sampling

Over the period of one year, a total of 150 Gram-negative isolates were tested. All isolates were identified based on colony morphology, Gram staining, and biochemical tests (Triple sugar iron agar, Citrate utilization test, Oxidase test, and Urease test) (Remel, Lenexa, KS, USA), followed by confirmation using the Vitek 2 system (Biomérieux, Marcy l’Etoile, France) according to the manufacturer’s instructions.

### 2.4. Antimicrobial Susceptibility Testing

Antimicrobial susceptibility testing was performed according to Clinical and Laboratory Standards Institute recommendations (M100 document) [16]. The diffusion method on Mueller–Hinton agar was used to test susceptibility to different antibiotics, including amoxicillin (AMX) (30 µg), cefipime (FEP) (30 µg), cephalothin (CF) (30 µg), ceftazidime (30 µg), amoxicillin-clavulanic acid (AMC) (30 µg), cefotaxime (CTX) (30 µg), ertapenem (ETP) (10 µg), aztreonam (30µg), meropenem (MEM) (10 µg), and imipenem (IPM) (10 µg) (Oxoid, Basingstoke, UK).

The selected isolates were resistant to ertapenem (≤18 mm), imipenem (≤19 mm), and/or meropenem (≤19 mm). The resistant isolates were confirmed by the Vitek 2 system (Biomérieux, Marcy l’Etoile, France) at MIC values ≥ 2 µg/mL for ETP and ≥4 µg/mL for IPM and MEM.

### 2.5. Polymerase Chain Reaction (PCR) for Detection of Carbapenemase Genes

PCR testing was performed for the detection of different carbapenemase genes (*bla_NDM_*, *bla_KPC_*, *bla_IMP_*, and *bla_VIM_*) as follows.

### 2.6. DNA Extraction

Total DNA from all isolates was extracted using the boiling lysis method. This was carried out by picking 3 representative colonies of the same morphological type from the slants of the isolated and identified bacteria, then transferring them into a tube containing 3 mL of Tryptic Soy Broth (TSB) (Remel, Lenexa, KS, USA), and incubating them at 37 °C for 18 h. An amount of 1 mL of the overnight bacterial culture was centrifuged at 8000× *g* for 2 min, and then sediment was washed with nuclease-free water, homogenized, and added to 0.1 mL distilled water and heated for 15 min at 95 °C.

Lysate preparations were clarified by centrifugation (typically 15,000× *g* for 10 min). The clarified supernatant was transferred to a clean 1.5 mL Eppendorf tube. The isolated DNAs from boiled lysates were checked for quality and purity by Nanodrop (ThermoFisher Scientific, Waltham, USA); good-quality DNA had an A260/A280 ratio of 1.8–2.0. All DNA samples were stored at −80 °C until used for gene detection.

### 2.7. Amplification of Carbapenemase Genes

Uniplex PCR reactions were performed with the following primer pairs. For *bla_KPC_* which belongs to class A serine enzymes: F 5′-CGTTGACGCCCAATCC-3′ and R 5′-ACCGCTGGCAGCTGG-3′ to amplify 390 bp fragment [17,18]. For *bla_IMP_* which belongs to class B metallo-b-lactamases (MBLs): F 5′-CATGGTTTGGTGGTTCTTGT-3′ and R 5′-ATAATTTGGCGGACTTTGGC-3′ to amplify 488 bp fragment [17,18]. For *bla_VIM_* which belongs to class B metallo-b-lactamases (MBLs): F 5′-ATTGGTCTATTTGACCGCGTC-3′ and 5′-TGCTACTCAACGACTGAGCG-3′ that amplify 780 bp fragment [17,18]. Lastly, for *bla_NDM-1_* (New Delhi metallo-b-lactamase-1): F 5′-GGTTTGGCGATCTGGTTTTC-3′ and R 5′-CGGAATGGCTCATCACGATC-3′ to amplify 621 bp fragment (Table 1) [19].

Amounts of 50 ng of DNA, 0.25 pM of each forward and reverse primer, 2 µL of Taq polymerase in a buffer containing 100 mM Tris-HCl, 1.5 mM of MgCl_2_ (pH 8.3) and 500 mM KCl, and 40 µM of deoxynucleotide triphosphate (dNTPs) were used. To obtain this concentration, a mix of 18 µL 2x Taq PCR master mix, 4.5 µL distilled water, and 0.25 µL of each forward and reverse primers was added to 2 µL of supernatant containing DNA used as the template for the PCR reaction (a total volume of 25 µL). Perkin-Elmer’s thermal cycler (Applied Biosystems, Waltham, MA, USA) was programmed for pre-denaturation at 95 °C for 5 min, 35 cycles of reaction at 95 °C for 45 s for denaturation, 48 °C for 30 s with *bla_KPC_*, 52 °C for *bla_IMP_*, *bla_VIM_*, and *bla_NDM_* for annealing and extension at 72 °C for 45 s, and final extension at 72 °C for 7 min. The amplified product was electrophoresed after application of ethidium-bromide-stained 1.5% agarose gel for the detection of specific bp products for each gene.

### 2.8. PCR-Based Replicon Typing (PBRT) of the Plasmid

This kit is a set of 8 specific standard PCR assays optimized to perform 8 multiplex PCRs for the amplification of 25 replicons, HI1, HI2, I1, I2, X1, X2, L/M, N, FIA, FIB, FIC, FII, FIIS, FIIK, W, Y, P, A/C, T, K, U, R, B/O, HIB-M, and FIB-M, representative of major plasmid incompatibility groups and replicase genes identified on resistance plasmids circulating among Gram-negative bacteria (Diatheva, Fano, Italy) [11,12,13,14]. Positive controls for all the respective replicons were included in the test (Table 2).

For PBRT, total DNA was obtained by the boiling lysis method according to the manufacturer’s instructions [20]. DNA purification was carried out by the Wizard Genomic DNA System Promega Purification Kit (Promega Corporation, Madison, WI, USA). The quality and purity of DNA templates were checked by Nanodrop (ThermoFisher Scientific, Waltham, MA, USA) as previously described. The cycling parameters were 10 min of denaturation at 95 °C, followed by 25–30 cycles at 60 °C for annealing (30 s each), and then 5 min of extension at 72 °C by Perkin-Elmer’s thermal cycler (Applied Biosystems, Waltham, MA, USA). The PBRT Amplification mixes were thawed, vortexed for 20 s, and centrifuged briefly. In 8 separate sterile 1.5 mL vials, the amplification reaction mixes (M1–M8) were prepared for each DNA sample or control by mixing 23.8 µL of each mix with 0.2 µL DNA polymerase. Each vial was mixed for 20 s and was centrifuged briefly. The DNA samples or PBRT positive controls (1 µL) were added to this 24 µL amplification mix aliquot and vortexed briefly. Following this, 5 µL of DNA loading buffer was added directly to amplified samples, and 5 µL of amplicons was loaded on a 2.5% agarose gel containing ethidium bromide in the presence of a DNA standard specific for the low range (100–1000 bp).

### 2.9. Statistical Analysis

After anonymizing all clinical and demographic inpatient data, the statistical analysis was performed. Data were entered and analyzed using IBM SPSS Statistics for Windows, Version 25.0. Armonk, NY, USA: IBM Corp. Quantitative data were expressed as frequency and percentage. The Chi-Square test (or Fisher’s exact test) was used for qualitative data from two groups. For qualitative data from more than two groups, the Chi-Square test with the Bonferroni method was used. For any of the used tests, results were considered statistically significant if the *p*-value was ≤0.05.

## 3. Results

Among 150 isolates, only 30 (20%) demonstrated carbapenem resistance. Out of the 30 isolates, 10 (33.3%) were *Klebsiella pneumoniae*, 8 (26.7%) were *Escherichia coli*, 5 (16.7%) were *Enterobacter cloacae*, 4 (13.3%) were *Pseudomonas aeruginosa*, and 3 (10%) were *Citrobacter freundii*. *Klebsiella pneumoniae* was the most frequent carbapenem-resistant organism isolated from wounds. Positive cultures were mostly obtained from cutaneous wound aspirates, blood cultures, catheter tips, and sputum. For more details, see Table 3.

The frequency of carbapenemase-encoding genes in tested organisms is shown in Table 4; *bla_NDM_ 15* (50%) was the highest, followed by *bla_VIM_* 9 (30%), *bla_IMP_* 8 (26.7%), and *bla_KPC_* 7 (23.3%). Out of the 30 tested isolates, 21 harbored at least one of the carbapenemase-producing genes; however, there was no statistically significant difference in the distribution of genes in different organisms. *Klebsiella pneumoniae* had the most copies of the *bla_NDM_* gene (six isolates), followed by *Escherichia coli* (five isolates). However, nine isolates, including three *Klebsiella pneumoniae*, two *Enterobacter cloacae*, two *Pseudomonas aeruginosa*, one *Citrobacter freundii*, and one *Escherichia coli*, did not have any of these genes.

Some isolates exhibited a heterogeneous pattern of carbapenemase-encoding genes, ranging from dual positivity (six isolates) to triple positivity (three isolates), while quadruple positivity was detected in two isolates (one *E. coli* and one *Enterobacter cloacae*).

Plasmids were detected in 25 isolates (10 *Klebsiella pneumonia,* 8 *Escherichia coli, 5 Enterobacter cloacae,* and 2 *Pseudomonas aeruginosa* isolates (83.3%)), with a statistically significant difference (*p*-value = 0.01). On the other hand, samples with no detectable plasmids were three *Citrobacter freundii* (60%) and two *Pseudomonas aeruginosa* (40%).

Plasmid replicons were found more often in *Escherichia coli*, *Enterobacter cloacae*, and *Klebsiella pneumoniae* than in *Citrobacter freundii*; however, this difference was not statistically significant when compared to *Pseudomonas aeruginosa*. In *Klebsiella pneumonia* isolates, the most common plasmid replicons were I1α in 5/10 (50%) isolates, FIIK in 4/10 (40%) isolates, and A/C, FIB, P, and R in 3/10 (30%) isolates each. Among *Escherichia coli* isolates, replicons A/C, FIIK, I1 α, L, and R were found three out of eight (37.5%) of isolates, whereas replicons FIA and FII (two out of five, 40%) and FIB (two out of four, 50%) were most abundant among *Enterobacter cloacae* and *Pseudomonas* (Table 5).

An analysis of the data in Table 6 revealed that eight isolates had a single replicon (two *Klebsiella pneumoniae*, three *Escherichia coli,* and three *Enterobacter cloacae*). On the other hand, two replicons were detected in four *Klebsiella pneumoniae* isolates. Remarkably, polyreplicons were found in 13 isolates, with plasmid replicons ranging from 3 to 8. The most frequent replicons detected were FIB, I1α, and FIIK in eight isolates, followed by FIA and R in seven isolates each.

## 4. Discussion

Multidrug-resistant (MDR) and extensively drug-resistant (XDR) Gram-negative bacteria infections are critical challenges for public health institutes due to limited antibiotic choices and high mortality [21,22,23]. This study provides an overview of how common carbapenemase-encoding genes and major plasmid incompatibility groups are in hospital-based Gram-negative isolates from Egypt. This could help set up an effective local policy for antibiotics, thus controlling the mounting problem of antimicrobial resistance.

In our study, 20% of the isolates were carbapenem resistant. *Klebsiella pneumoniae* and *Escherichia coli* topped the list of carbapenem-resistant bacteria. In line with our findings, Raheel et al. described that 34.1% of the isolated *Enterobacteriaceae* were carbapenem resistant [24]. Similar resistance patterns were also reported by Khattab et al., in which carbapenem-resistant *K. pneumoniae* and *Escherichia coli* were isolated at rates of 47.6% and 28.6%, respectively [14].

Interestingly, when we looked at the prevalence of different carbapenemase genes, *bla_NDM_* was the most frequently detected in 15 isolates. The solidity of our results was also confirmed by a 2-year study focused on *Enterobacteriaceae* isolates in 40 countries around the world, in which *bla_NDM_* was the most common gene (36.8%, 60/163) linked to carbapenem resistance [25]. Additionally, in a cross-sectional study at Tikur Anbessa Specialized Hospital, Addis Ababa, Ethiopia, out of 39 carbapenemase-producing *K*. *pneumoniae* isolates, *bla_NDM_* was the most dominant gene (92.9%) [26].

Focusing on Egypt, similar findings were reported in a study by El-Kholy and her colleagues, in which *bla_NDM_*, *bla_KPC_*, and *bla_VIM_* were the most common resistance genes in patients with surgical site infections [27]; *bla_KPC_* was also found in 13.6% of isolates in a recent study at Ain Shams University Hospital [28]. The widespread dissemination of carbapenem resistance in our locality can be explained by the irrational use of carbapenems and the lack of a national antibiotic stewardship program.

To our surprise, we detected a higher prevalence of concurrent multiple carbapenemases, namely dual positivity in six isolates, triple positivity in three isolates, and quadruple positivity in two isolates. In accordance with our results, multidrug resistance strains are continuously reported worldwide [29,30], and they are sustained by several mechanisms, such as R plasmids or transposons [31].

Remarkably, we detected plasmid replicons in 25 isolates (83.3%), prevalently carried by *Escherichia coli*, *Enterobacter cloacae,* and *Klebsiella pneumoniae*. In coherence with the findings of Zharikova et al., the absence of plasmid replicons in *Citrobacter freundii* isolates may be explained by the small plasmid size (2–15 kb) that was hardly detected by our method [32]

Remarkably, polyreplicons were found in 13 isolates, with plasmid replicons ranging from 3 to 8. The most frequent replicons detected were FIB, I1α, and FIIK in eight isolates, followed by FIA and R in seven isolates each. Moreover, InF (FIA, FIB, and FII) multireplicon plasmids in different combinations with other plasmids were detected in this study. In the literature, replicons F, A/C, and I1α are regarded as the main plasmid families associated with the spread of resistance genes in isolated *Enterobacteriaceae* worldwide [33]. A recent study by Castanheira et al. performed in the US found that 49.6% of the isolated *Enterobacteriaceae* were carbapenem resistant, with one *Klebsiella pneumoniae* carrying multireplicon plasmids (Inc types A/C and FII) [34]. The implications of these findings for the future of MDR may be quite concerning.

In line with previously published Chinese and European reports, we found that the IncF plasmid was prevalent in carbapenem-resistant *Enterobacteriaceae* [35,36]. IncF was also reported as the prevalent plasmid in animals, sustaining the idea of a potential interspecies influence in the global spread of MDR [37].

In some organisms (three isolates), there were no detected genes and no plasmid replicons, with the possibility of different resistance mechanisms other than carbapenemase production. On the other hand, some organisms expressed resistance genes without detectable plasmids (two isolates), with the possibility of the presence of a small plasmid or the presence of these genes on the bacterial chromosome. In addition, there were some organisms with plasmids detected but no resistance genes detected (six isolates), with the possibility of the presence of genes other than those tested in this study.

Evaluating in greater detail the replicon prevalence in different Enterobacteriaceae, *Klebsiella pneumoniae* had a 50% prevalence of Inc I1α, 40% of Inc FIIK, 30% of Inc A/C, 30% of FIB, 30% of P, and 30% of R. *Escherichia coli*, on the other hand, had a comparable plasmid replicon pattern with 37.5% Inc A/C, 37.5% FIIK, 37.5% I1, 37.5% L, and 37.5% R.

Conversely, Johnson et al. described the Inc FIB plasmid as the most frequent plasmid type linked to the spread of several ESBL genes in *Escherichia coli* isolates [38]. In the same line, Cao et al. reported that Inc FII was the predominant plasmid replicon in *Klebsiella* isolates (18/27, 66.7%). Interestingly, we reported infrequent plasmid amplicons, such as IncY, in accordance with some previous studies [24,39].

As regards the association between carbapenemase genes and plasmid replicons, we found an *Escherichia coli* isolate displaying both the *bla_NDM_* gene and the IncA/C plasmid replicon. Pal et al. also reported an association between the *bla_NDM-7_* gene and the IncX3 plasmid in multidrug-resistant *Escherichia coli* isolates from the Arabian Peninsula [40].

This article presents several strengths but also has some limitations connected with the study design (single year) and the heterogeneity of the samples; at the same time, it could also be regarded as a starting point for a more extensive yearly evaluation of the MDR population in our locality.

## 5. Conclusions

Our study demonstrated an alarming rise of plasmid-mediated carbapenem-resistant bacteria in our locality. The coexistence of resistance genes and plasmids highlights the importance of a targeted antibiotic surveillance program and the development of alternative therapeutic options at the local and international levels. Based on our results, we suggest a large-scale study with more *Enterobacteriaceae* isolates, testing other carbapenemase-encoding genes, and comparing the replicon typing method with other plasmid detection methods. We also recommend a national action plan to control the irrational use of antibiotics in Egypt.

## Figures and Tables

**Table 1:** List of the primers used for amplification of carbapenemase genes in the isolated *Enterobacteriaceae*.

Target Gene		Amplified Product (bp)	Target Amplicon
*bla_KPC_* [17,18] *F* *R*	5′-CGTTGACGCCCAATCC-3′ 5′-ACCGCTGGCAGCTGG-3′	390	Class A serine enzymes
*bla_IMP_* [17,18] *F* *R*	5′-CATGGTTTGGTGGTTCTTG -3′ 5′-ATAATTTGGCGGACTTTGGC-3′	488	Class B metallo-b-lactamases (MBLs)
*bla_VIM_* [17,18] *F* *R*	5′-ATTGGTCTATTTGACCGCGTC-3′ 5′-TGCTACTCAACGACTGAGCG-3′	780	
*bla_NDM-1_* [19] *F* *R*	5′-GGTTTGGCGATCTGGTTTTC-3′ 5′-CGGAATGGCTCATCACGATC-3′	621	New Delhi metallo-b- lactamase-1

**Table 2:** Target sites for PBRT Amplification mixes.

PCR Mix	M1	M2	M3	M4	M5	M6	M7	M8
**Target site** **(Amplicon length, bp)**	HI 1 (534) HI2 (298–308) I1α (159)	M (741) N (514) I2 (316) BO (159)	FIB (683) FIA (462) W (242)	L (854) P (534) X3 (284) I1γ (161)	T (750) A/C (418) FIIS (259–260)	U (843) X1 (370) R (251) FIIK (142–148)	Y (765) X2 (376) FIC (262) K (160)	HIB-M (570) FIB-M (440) FII (258–262)

**Table 3:** Frequency of organisms isolated from different samples.

Sample	Organism						χ^2^	*p*
	*K. pneumoniae*	*E. coli*	*Ent. cloacae*	*P. aeruginosa*	*C. freundii*	Total		
Cutaneous Wound aspirate	6 (60%)	4 (50%)	2 (40%)	2 (50%)	0 (0%)	14	26.524	0.622
Blood culture	1 (10%)	0 (0%)	1 (20%)	1 (25%)	1 (33.3%)	4		
Catheter tip	1 (10%)	1 (12.5%)	0 (0%)	0 (0%)	1 (33.3%)	3		
Sputum	1 (10%)	0 (0%)	1 (20%)	0 (0%)	1 (33.3%)	3		
Peritoneal fluid	0 (0%)	1 (12.5%)	0 (0%)	1 (25%)	0 (0%)	2		
Urine	0 (0%)	2 (25%)	0 (0%)	0 (0%)	0 (0%)	2		
Drainage fluid	0 (0%)	0 (0%)	1 (20%)	0 (0%)	0 (0%)	1		
Endotracheal tube	1 (10%)	0 (0%)	0 (0%)	0 (0%)	0 (0%)	1		
Total	10 (33.3%)	8 (26.7%)	5 (16.7%)	4 (13.3%)	3 (10%)	30		

**Table 4:** Frequency of carbapenemase-encoding genes in different organisms.

Resistance Genes	*K. pneumoniae*	*C. freundii*	*E. coli*	*E. cloacae*	*P. aeruginosa*	Total	χ^2^	*p*
*bla_IMP_*	1/10(10%)	1/3 (33.3%)	2/8(25%)	3/5 (60%)	1/4(25%)	8/30(26.7%)	4.347	0.412
*bla_VIM_*	1/10 (10%)	1/3 (33.3%)	4/8(50%)	3/5(60%)	0/4 (0%)	9/30(30%)	7.302	0.128
*bla_NDM-1_*	6/10 (60%)	2/3(66.7%)	5/8(62.5%)	1/5(20%)	1/4(25%)	15/30(50%)	4.033	0.457
*bla_KPC_*	2/10 (20%)	0/3(0%)	3/8 (37.5)	2/5 (40%)	0/4(0%)	7/30(23.3%)	3.866	0.470

**Table 5:** Percentage of replicons detected in different organisms.

Replicon	Total Frequency	Frequency per Organism					χ^2^	*p*-Value *
		*K. pneumoniae*	*E. Coli*	*E. cloacae*	*P. aeruginosa*	*C. freundii*		
A/C	6	3 (50%) a	3 (37.5%) a	0 (0%) a	0 (0%) a	0(0%) a	3.207	0.388
FIA	7	2 (20%) a	2 (25%) a	2 (40%) a	1 (50%) a	0(0%) a	1.190	0.932
FIB	8	3 (30%) a	2 (25%) a	1 (20%) a	2 (100%) a	0(0%) a	4.779	0.218
FII	6	1 (10%) a	2 (25%) a	2 (40%) a	1 (50%) a	0(0%) a	2.522	0.541
FIIK	8	4 (40%) a	3 (37.5%) a	1 (20%) a	0 (0%) a	0(0%) a	1.677	0.657
FIIS	2	0 (0%) a	0 (0%) a	1 (20%) a	1 (50%) a	0(0%) a	7.337	0.071
HI2	1	1 (10%) a	0 (0%) a	0 (0%) a	0 (0%) a	0(0%) a	1.563	1.000
I1α	8	5 (50%) a	3 (37.5%) a	0 (0%) a	0 (0%) a	0(0%) a	4.894	0.188
L	5	1 (10%) a	3 (37.5%) a	0 (0%) a	1 (50%) a	0(0%) a	4.531	0.209
FIB-M	3	1 (10%) a	2 (25%) a	0 (0%) a	0 (0%) a	0(0%) a	2.273	0.673
P	5	3 (30%) a	1 (12.5%) a	0 (0%) a	1 (50%) a	0(0%) a	3.281	0.387
R	7	3 (30%) a	3 (37.5%) a	1 (20%) a	0 (0%) a	0(0%) a	1.314	0.804
X1	5	1 (10%) a	2 (25%) a	1 (20%) a	1 (50%) a	0(0%) a	1.875	0.727
X2	1	0 (0%) a	0 (0%) a	1 (20%) a	0 (0%) a	0(0%) a	4.167	0.285
X3	1	1 (10%) a	0 (0%) a	0 (0%) a	0 (0%) a	0(0%) a	1.563	1.000
Y	2	0 (0%) a	1 (12.5%) a	1 (20%) a	0 (0%) a	0(0%) a	2.242	0.567
Total replicon detected	25	10 (100%) a	8 (100%) a	5 (100%) a	2 (50%) a,b	0 (0%) b	22.8	<0.0005

* The *p*-values by Chi-Square test (Monte Carlo significance) comparing the frequency of detected versus non-detected replicons. Similar small letters ‘a’ and ‘b’ indicate no significant difference, while different letters indicate a significant difference.

**Table 6:** Patterns of carbapenemase-encoding genes and plasmid replicons detected in studied isolates.

Organism	Genes Detected	Plasmid Replicon Detected
*Klebsiella pneumoniae*	^_^	I1α FIIK
*Klebsiella pneumoniae*	*bla_NDM_*	FIB
*Klebsiella pneumoniae*	*bla_NDM_*	FIIK
*Klebsiella pneumoniae*	*bla_VIM_ bla_NDM_*	I1α X1
*Klebsiella pneumoniae*	*^_^*	FIA FIB
*Klebsiella pneumoniae*	*bla_IMP_ bla_NDM_*	A/C X3
*Klebsiella pneumoniae*	*bla_NDM_*	FIA FII FIIK I1α FIB-M R
*Klebsiella pneumoniae*	*bla_KPC_*	A/C HI2 I1α P R
*Klebsiella pneumoniae*	*^_^*	A/C FIB L P
*Klebsiella pneumoniae*	*bla_NDM_ bla_KPC_*	FIIK I1α P R
*Escherichia coli*	*bla_VIM_ bla_KPC_*	L, FIB-M, R X1
*Escherichia coli*	*bla_NDM_*	A/C FIIK I1α R X1
*Escherichia coli*	*^_^*	A/C FIIK L
*Escherichia coli*	*bla_NDM_*	FIA FIB FII L R
*Escherichia coli*	*bla_IMP_ bla _VIM_ bla_NDM_ bla_KPC_*	A/C FIA FIB FII I1α FIB-M P Y
*Escherichia coli*	*bla_IMP_ bla_VIM_ bla_NDM_*	I1α
*Escherichia coli*	*bla_VIM_*	L
*Escherichia coli*	*bla_NDM_ bla_KPC_*	FIIK
*Enterobacter cloacae*	*^_^*	Y
*Enterobacter cloacae*	*bla_IMP_ bla_VIM_ bla_KPC_*	FIA FII X2
*Enterobacter cloacae*	^_^	X1
*Enterobacter cloacae*	*bla_IMP_ bla_VIM_ bla_NDM_ bla_KPC_*	FIA FIB FII FIIS R
*Enterobacter cloacae*	*bla_IMP_ bla_VIM_*	FIIK
*Pseudomonas aeruginosa*	*bla_NDM_*	FIA FIB FII FIIS
*Pseudomonas aeruginosa*	*bla_IMP_*	FIB L P X1
*Pseudomonas aeruginosa*	*-*	-
*Pseudomonas aeruginosa*	*-*	-
*Citrobacter freundii*	*-*	-
*Citrobacter freundii*	*bla_NDM_*	-
*Citrobacter freundii*	*bla_IMP_ bla_VIM_ bla_NDM_*	-

## Data Availability

The data presented in this study are available on request from the corresponding author. The data are not publicly available due to privacy restrictions.
